# Composition of general practices in Western countries and associations with their perceived ability to manage patients with chronic conditions: a secondary analysis of survey data

**DOI:** 10.3399/BJGPO.2024.0200

**Published:** 2026-02-25

**Authors:** Adeline Cachou de Camaret, Pascal Wild, Nicolas Senn, Christine Cohidon

**Affiliations:** 1 Department of Family Medicine, Centre of Primary Care and Public Health (Unisanté), University of Lausanne, Lausanne, Switzerland; 2 PW Statistical Consulting, Laxou, France

**Keywords:** primary care team, chronic disease, model of care, primary health care, quality of health care, delivery of health care

## Abstract

**Background:**

Primary care teams (PCTs) are recognised to improve the quality of care. However, few studies have examined which PCT composition best meets patients’ primary care (PC) needs.

**Aim:**

To describe the composition of PCTs in 11 Western countries and investigate potential associations with GPs’ opinions about their practice’s ability to manage patients with chronic conditions and communicate with caregivers.

**Design & setting:**

A secondary analysis of the data from 11 Western countries that participated in the 2019 Commonwealth Fund International Health Policy Survey of Primary Care Physicians was conducted.

**Method:**

A hierarchical clustering algorithm was used to characterise different types of PCT according to the composition of the healthcare professionals (HCPs) making them up, in addition to GPs. Associations between practice types and two GP-reported indicators were subsequently assessed: their practice’s coordination with social services (SS) and other community providers (CPs); and ability to manage patients with chronic conditions.

**Results:**

Overall, 13 200 responses were analysed. Five types of PCT were characterised (traditional, multidisciplinary, nurse-centred, psychologist-centred, and physiotherapist-centred). The traditional type represented 51.6% of all PCTs; they were mainly composed of moderate percentages of all the HCPs. The multidisciplinary type (11.9%) were composed of high percentages of the different HCPs. After controlling for country, the multidisciplinary type reported better coordination with SS and CPs than did traditional ones (odds ratio 0.39, 95% confidence interval = 0.29 to 0.53).

**Conclusion:**

Multidisciplinary PCTs reported better outcomes than traditional ones regarding their coordination with SS and CPs, and perceived ability to manage patients with chronic conditions. These results should encourage governmental efforts to promote PC that uses multidisciplinary PCTs.

## How this fits in

If primary care teams (PCTs) are recognised to improve the quality of care, few studies have examined which PCT composition best meets patients’ primary care needs. The results of the present study show that multidisciplinary practices (composed of high percentages of the different healthcare professionals [HCPs]) reported better coordination with social services (SS) and other community providers (CPs), and greater ability to manage patients with chronic conditions than traditional ones (GP-centred model). These results should encourage GPs to move towards this type of organisational model, as well as encourage governmental efforts to promote a primary care that uses multidisciplinary PCTs.

## Introduction

Populations are ageing rapidly in Western countries, contributing to an increased prevalence of chronic diseases and multimorbidity.^1–3^ Using integrated care, defined as *‘patient-centred, proactive, and well-coordinated care, making use of innovative technologies to support patient’s self-management and improving multidisciplinary collaboration between caregivers’*,^4,5^ seems to be an effective means of ensuring accessible, cost-effective, high-quality care,^6^ especially for patients with multiple chronic conditions.^7^ GPs, working within communities, appear to be very well positioned to manage the coordination of care;^8^ however, the shortage of physicians in industrialised countries demonstrates the urgent need to develop new models of primary care (PC).^9,10^ This context has led to the emergence of new concepts such as ‘shared care plans’^11^ and new roles such as ‘case managers’.^12,13^

Developing interprofessional teams of HCPs appears to be an appropriate option for implementing patient-centred care reforms without overloading GPs.^14^ Some studies have shown that developing patient-centred medical homes (PCMHs) have improved the quality of care^15^ and interprofessional collaboration,^16,17^ and reduced costs,^18–20^ levels of clinician burnout,^21,22^ and emergency department use.^23^ Furthermore, patient co-management between physicians and advanced practice nurses (APNs) was demonstrated to improve clinical patient outcomes,^24^ potentially save costs,^25^ and alleviate individual clinician workloads.^26^ Integrating pharmacists, nutritionists, psychologists, and SS into PCTs increased the quality of care and improved patient outcomes.^27–33^

Although PCMHs and similar types of PC organisation have been described as models of care delivery that could improve the quality of care and reduce costs,^14^ many PC practices in Western countries (for example, Switzerland) still work with traditional staffing models, that is, GPs working single-handedly with limited or no multidisciplinary collaboration.^34–36^

The present study aimed to describe the composition of PCTs in 11 Western countries and investigate whether different types of PCT were associated with GPs’ views about their practice’s ability to manage patients with chronic conditions. According to the arguments discussed above, we hypothesised that multidisciplinary management of patients with chronic disease would be perceived as being of better quality by GPs.

## Method

### Commonwealth Fund international surveys

We extracted data from the 2019 Commonwealth Fund International Health Policy Survey of Primary Care Physicians. For more than two decades, the Commonwealth Fund has been conducting international surveys of nationally representative random samples of 13 200 PC physicians regarding their practice’s ability to manage the care of patients with complex needs, to communicate with other medical specialties and community-based providers, and to use health information technology.^37^ Australia, Canada, France, Germany, the Netherlands, New Zealand, Norway, Sweden, Switzerland, the UK, and the US participated in the 2019 edition.^38,39^ This multi-country study allows the inclusion of a wide variety of healthcare systems between different countries, particulary in terms of governance, financing, and cultural aspects.

### Population and data collection

The 2019 survey’s study protocol has already been detailed elsewhere;^38,39^ thus, we only present its main characteristics relevant to our study. National samples of physicians were randomly selected from government or private lists of PC physicians, or for France, from a nationally representative panel of PC physicians.^38^ Experts in each country defined the medical specialties responsible for PC, recognising that roles, training, and scopes of practice varied across countries. GPs or family physicians were included in every country, with internists and paediatricians also sampled in Germany, Switzerland, and the US.^38^

Physicians were surveyed between January and June 2019 by post, via the internet, or by telephone, according to each country’s best practices for reaching physicians and maximising response rates.^38,39^ Data were weighted to align with national benchmarks for including key geographic and demographic aspects (sex, age, region, type of area, and medical specialty).^38^

### Data

The questionnaire was designed by experts in research surveys, pretested in each country, adjusted with country-specific wording and translated as required to ensure comparability across countries. To investigate the composition of PCTs, we used the following question: *‘In your main practice, do the following healthcare professionals work on your team to provide care for your patients? a. Nurse(s), b. Advanced practice nurse(s) (for example, nurse practitioner[s]), c. Physician(s) or medical assistant(s), d. Nutritionist(s) or dietician(s), e. Pharmacist(s), f. Psychologist(s) or mental health professional(s), g. Physical therapist(s) or physiotherapist(s), h. Social worker(s).’* Response options were ‘Yes’ or ‘No’.

We then selected data from a question about coordination with CPs: *‘Do you or any other HCP working with you in your practice coordinate care with social services or other community providers?’* Response options were ‘Yes, frequently’, ‘Yes, occasionally’, or ‘Never’. We also included data from a question about the care management of chronic conditions: *‘How prepared is your practice, with respect to having sufficient skills and experience, to manage care for patients with chronic conditions?’* Response options were ‘Well prepared’, ‘Somewhat prepared’, and ‘Not prepared’.

Finally, we also selected variables for use as confounding factors in our logistic regressions. These variables included each country’s physicians’ sociodemographic characteristics (sex and age), practice location (rural or urban area), consultation durations, and communication habits with patients and other HCPs.

### Statistical analysis

All statistical analyses were performed using Stata software (version 14.1). First, we used a hierarchical clustering algorithm, with complete linkage and a simple matching similarity coefficient, to characterise the different types of PCTs in the 11 countries, according to the composition of the HCPs making them up, in addition to GPs. The clusters were then interpreted and denominated according to the types of HCPs identified. We described the PCT types in each country by describing the distribution and frequencies of different kinds of HCPs. Second, we modelled the dependent variables of communication with CPs and ability to manage the care of patients with chronic conditions in logistic regressions as a function of selected physician and practice characteristics. The two dependent variables were dichotomised as follows: 1) regarding the coordination with SS and CPs, we modelled poor coordination with SS and other CPs (response ‘Never’, coded as 1) versus good coordination (grouping the responses ‘Yes, frequently’ and ‘Yes, occasionally’, coded as 0); and 2) regarding the ability to manage patients with chronic disease, we modelled poor ability (response ‘Not prepared’, coded as 1) versus good ability (grouping the responses ‘Somewhat prepared’ and ‘Well prepared’, coded as 0). For both dependent variables, each independent variable was introduced separately and, in a second stage, jointly, first without adjusting for the country (final model 1), and second, adjusting for the country (final model 2).

## Results

### Characteristics of the sample

Table 1 presents the main characteristics of the sample of responders by country. The questionnaire was completed by 13 200 GPs, representing a response rate ranging from 14.5% (Australia) to 48.7% (the Netherlands), with an overall average response rate of 29.1%. The proportion of female GPs was 46.0% (varying from 37.5%–55.0%). The proportion of GPs aged ≤44 years was 34.0% (varying from 1.7%–57.5%). Finally, GPs aged ≥65 years represented 13.7% (varying from 2.1%–17.9%).

**Table 1.** Responders’ general sociodemographic characteristics and practice compositions in the 11 countries included in the 2019 Commonwealth Fund International Health Policy Survey of Primary Care Physicians (weighted data)

Category	Australia	Canada	France	Germany	Netherlands	New Zealand	Norway	Sweden	Switzerland	UK	US	Total (*n*) and average (%)
**Participants, *n***	500	2569	1287	809	788	503	661	2411	1095	1001	1576	13 200
**Response rate, %**	14.5	39.3	20.0	14.7	48.7	16.2	33.8	42.2	42.8	26.8	21.2	29.1
**Sex, %**												
Male	54.9	54.0	62.5	54.6	47.7	44.9	56.2	48.8	59.4	53.9	55.1	53.9
Female	45.1	45.9	37.5	45.3	52.2	55.0	43.7	51.1	40.5	46.0	44.8	46.0
**Age, years, %**												
≤44	39.0	34.2	19.2	1.7	40.8	32.3	46.8	39.6	20.3	57.5	30.8	34.0
45–64	46.8	50.3	62.7	66.5	56.9	55.5	44.2	47.4	62.2	36.2	51.3	52.2
≥65	14.2	15.3	17.9	16.7	2.1	12.1	8.8	12.9	17.4	6.2	17.7	13.7
**Percentage of practices including**												
Nurses	90.8	68.2	50.0	23.7	35.2	99.1	50.2	98.6	20.9	99.5	72.0	64.4
Advanced practice nurses	16.2	36.9	0	2.9	13.6	35.4	12.6	93.7	5.2	63.4	61.1	31.0
Medical assistants	3.2	11.7	0	97.5	96.1	14.3	97.6	25.6	90.8	12.8	38.6	44.4
Nutritionists	53.2	45.0	21.2	16.7	31.5	16.3	6.1	58.0	18.3	16.6	34.2	28.8
Pharmacists	32.9	52.1	0	1.7	25.6	51.3	6.3	19.3	2.8	73.1	35.0	27.3
Psychologists	57.8	43.3	31.0	6.4	94.9	36.3	16.5	96.3	15.3	31.5	35.8	42.3
Physiotherapists	44.3	28.5	37.1	4.7	21.0	31.2	22.2	76.3	11.4	26.1	25.7	29.9
Social workers	7.9	44.2	15.7	0.9	8.6	17.1	7.9	0	2.3	17.2	39.6	14.7

The non-physician HCPs most commonly present in practices were nurses (64.4%), followed by medical assistants (MAs; 44.4%). Other HCPs were present in varying percentages and with great variability depending on the country (Table 1).

### Practice types according to the composition of their HCPs

The cluster analysis identified five types of practice according to their HCP composition. To improve readability, we denominated these as follows: traditional, multidisciplinary, nurse-centred, psychologist-centred, and physiotherapist-centred PCTs.

**Figure 1.**
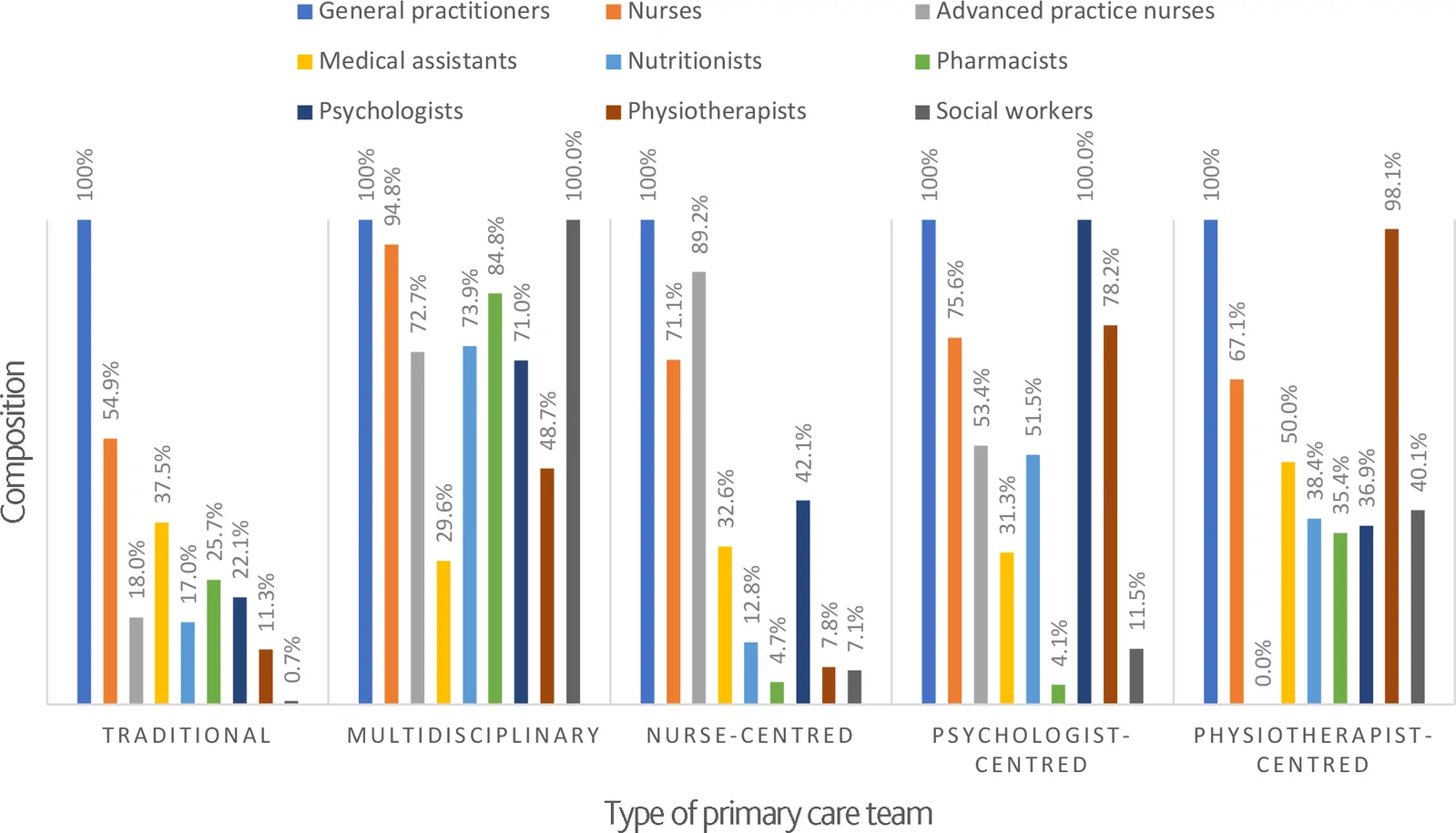
Characterisation of types of primary care team (PCT) according to their compositions of different healthcare professionals (in addition to GPs) (*N* = 13 200). Overall proportions of types of PCTs: traditional, 51.6%; multidisciplinary, 11.9%; nurse-centred, 11.9%; psychologist-centred, 18.7%; and physiotherapist-centred, 5.3%.

Traditional PCTs were the majority, making up 51.6% of practices internationally. In this kind of PCT, we found a vast majority of nurses (54.9%), followed by MAs (37.5%). Psychologist-centred PCTs were the second most common (18.7%), with every practice comprising psychologists. Multidisciplinary PCTs represented 11.9% of practices and were characterised by high proportions of all HCPs (94.8% comprised nurses, 100% social workers [SWs], 84.8% pharmacists, 73.9% nutritionists, 72.7% APNs, 48.7% physiotherapists, and 29.6% MAs). Nurse-centred PCTs, 11.9% of practices, were characterised by the significant presence of APNs (89.2%) and nurses (71.1%). Finally, physiotherapist-centred PCTs were the least represented type of practice (5.3%) (Figure 1).

**Figure 2.**
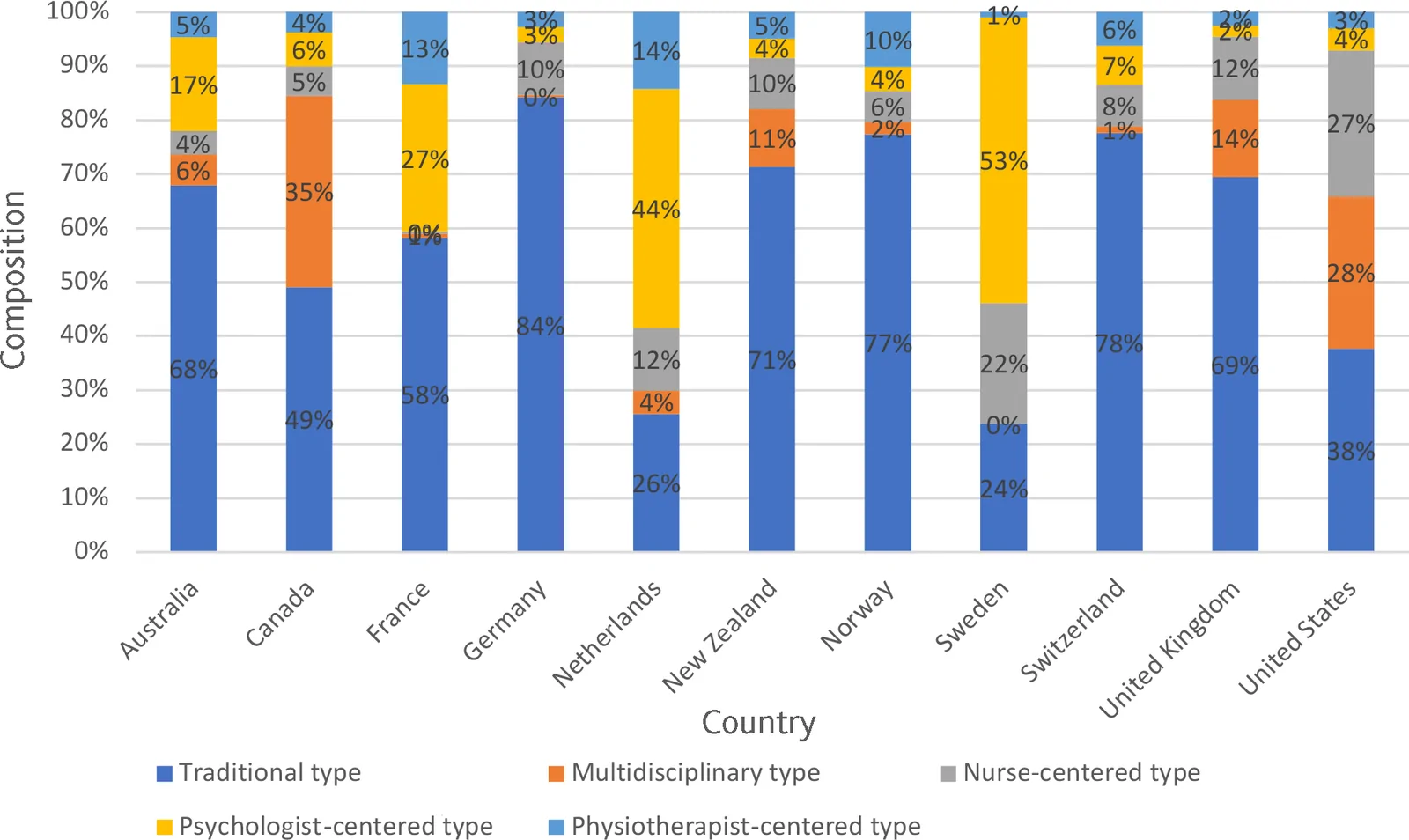
Types of primary care team by country (weighted data)

Traditional practices were highly represented in all the countries, varying from 24% (Sweden) to 84% (Germany). Multidisciplinary practices were mainly found in North America (35% in Canada and 28% in the US). Nurse-centred practices were mainly represented among US practices (27%) and psychologist-centred practices were mainly present in Sweden (53%) (Figure 2).

### Associations between practice types and coordination with social services and community providers

In the multivariate analyses, multidisciplinary practices were associated with better coordination with SS and CPs than were traditional practices (OR 0.42, 95% CI = 0.31 to 0.55). In comparison with those located in cities, significantly better coordination with SS and CPs was observed when practices were located in a small town (OR 0.69, 95% CI = 0.58 to 0.82) or a rural area (OR 0.53, 95% CI = 0.42 to 0.67) (Table 2 and Supplementary Table S1).

**Table 2.** Logistic regression of poor coordination with social services or other community providers as a function of physician and practice characteristics (*N* = 11 337)

		Univariate model			Multivariate model 1^a^			Multivariate model 2^b^		
**Category**	***n***	**OR**	**95% CI**	***P*** **value**	**OR**	**95% CI**	***P*** **value**	**OR**	**95% CI**	***P*** **value**
**Sex**										
Male (reference)	7072	1.00	—	—	1.00	—	—	1.00	—	—
Female	5948	1.03	0.92 to 1.15	0.602	0.95	0.83 to 1.08	0.449	0.95	0.83 to 1.09	0.455
**Age, years**										
≤44 (reference)	4376	1.00	—	—	1.00	—	—	1.00	—	—
45–54	3151	0.91	0.78 to 1.05	0.206	0.95	0.80 to 1.12	0.539	1.02	0.86 to 1.21	0.819
55–64	3815	0.93	0.81 to 1.07	0.309	1.00	0.85 to 1.17	0.990	1.04	0.88 to 1.23	0.627
≥65	1691	1.11	0.94 to 1.32	0.214	0.91	0.74 to 1.13	0.405	0.95	0.76 to 1.18	0.650
**Area**										
City (reference)	5590	1.00	—	—	1.00	—	—	1.00	—	—
Suburb	2676	0.99	0.86 to 1.13	0.902	0.97	0.83 to 1.14	0.712	1.01	0.85 to 1.19	0.902
Small town	2761	0.62	0.53 to 0.72	<0.001	0.69	0.58 to 0.82	<0.001	0.82	0.68 to 0.98	0.033
Rural	1788	0.43	0.35 to 0.53	<0.001	0.53	0.42 to 0.67	<0.001	0.69	0.54 to 0.88	0.002
**Communication with other HCPs**										
Yes, often (reference)	8125	1.00	—	—	1.00	—	—	1.00	—	—
Sometimes or rarely	3819	1.70	1.51 to 1.93	<0.001	1.80	1.58 to 2.05	<0.001	2.00	1.75 to 2.30	<0.001
Never	126	5.03	3.42 to 7.40	<0.001	4.78	3.16 to 7.23	<0.001	5.60	3.63 to 8.64	<0.001
**GP communicates using email**										
No (reference)	4677	1.00	—	—	1.00	—	—	1.00	—	—
Yes	8026	0.95	0.85 to 1.07	0.392	0.80	0.70 to 0.92	0.002	0.73	0.62 to 0.87	<0.001
**Mean consultation time**										
≥5 minutes to ≤10 minutes (reference)	2705	1.00	—	—	1.00	—	—	1.00	—	—
>10 minutes to ≤15 minutes	4243	1.32	1.11 to 1.58	0.001	1.49	1.21 to 1.82	<0.001	1.05	0.83 to 1.32	0.696
>15 minutes to ≤20 minutes	3290	1.75	1.47 to 2.09	<0.001	1.97	1.60 to 2.42	<0.001	1.00	0.77 to 1.30	0.986
>20 minutes to ≤90 minutes	2635	2.18	1.83 to 2.61	<0.001	2.38	1.91 to 2.95	<0.001	1.04	0.79 to 1.38	0.776
**Practice type**										
Traditional type (reference)	6721	1.00	—	—	1.00	—	—	1.00	—	—
Multidisciplinary	1641	0.50	0.39 to 0.62	<0.001	0.42	0.31 to 0.55	<0.001	0.39	0.29 to 0.53	<0.001
Nurse-centred	1543	1.65	1.41 to 1.93	<0.001	1.62	1.34 to 1.95	<0.001	1.11	0.91 to 1.37	0.298
Psychologist-centred	2427	1.54	1.35 to 1.77	<0.001	1.57	1.34 to 1.84	<0.001	0.88	0.74 to 1.05	0.173
Physiotherapist-centred	688	0.95	0.73 to 1.23	0.698	1.00	0.74 to 1.35	0.988	0.84	0.62 to 1.23	0.251
**Country**										
Australia (reference)		1.00	—	—	—	—	—	1.00	—	—
Canada		1.41	0.99 to 1.99	0.054	—	—	—	2.01	1.30 to 3.11	0.002
France		2.15	1.51 to 3.07	<0.001	—	—	—	3.77	2.44 to 5.82	<0.001
Germany		0.34	0.20 to 0.50	<0.001	—	—	—	0.28	0.13 to 0.60	0.001
The Netherlands		0.98	0.65 to 1.49	0.933	—	—	—	2.15	1.30 to 3.57	0.001
New Zealand		0.42	0.24 to 0.75	0.003	—	—	—	0.51	0.26 to 1.00	0.052
Norway		0.41	0.24 to 0.70	0.001	—	—	—	0.85	0.46 to 1.59	0.615
Sweden		3.04	2.17 to 4.26	<0.001	—	—	—	5.58	3.53 to 8.82	<0.001
Switzerland		0.73	0.48 to 1.10	0.130	—	—	—	0.71	0.41 to 1.24	0.230
UK		0.51	0.33 to 0.80	0.003	—	—	—	0.89	0.52 to 1.52	0.672
US		1.89	1.33 to 2.60	<0.001	—	—	—	2.74	1.73 to 4.33	<0.001

^a^Without adjusting for countries. ^b^Adjusting for individual countries. HCP = healthcare professional. OR = odds ratio.

When adjusted to individual countries, however, most of these results were no longer significant, apart from the better coordination with SS and CPs seen among multidisciplinary practices than among traditional ones (OR 0.39, 95% CI = 0.29 to 0.53), which stayed significant (Table 2).

### Associations between practice types and ability to manage chronic conditions

Physicians in multidisciplinary and nurse-centred practice types reported being better prepared for managing patients with chronic conditions than traditional ones (OR 0.70, 95% CI = 0.59 to 0.84 and OR 0.82, 95% CI = 0.69 to 0.97, respectively). Female GPs, GPs who communicated poorly (sometimes or rarely; never) with home-care services, and those whose consultations were longest all reported being less well prepared to deal with patients with chronic conditions. On the other hand, GPs practising in small towns or rural areas (versus city settings), and those used to communicating via email, reported being better prepared to manage patients with chronic conditions. When the model was adjusted to individual countries, most of these results were no longer significant (Table 3).

**Table 3.** Logistic regression of poor practices’ perceived ability to deal with patients with chronic conditions as a function of physician and practice characteristics (*N* = 11 376)

		Univariate model			Multivariate model 1^a^			Multivariate model 2^b^		
**Category**	***n***	**OR**	**95% CI**	***P*** **value**	**OR**	**95% CI**	***P*** **value**	**OR**	**95% CI**	***P*** **value**
**Sex**										
Male (reference)	7057	1.00	—	—	1.00	—	—	1.00	—	—
Female	5946	1.18	1.08 to 1.29	<0.001	1.12	1.01 to 1.24	0.027	1.15	1.03 to 1.28	0.013
**Age, years**										
≤44 (reference)	4362	1.00	—	—	1.00	—	—	1.00	—	—
45–54	3152	0.81	0.72 to 0.91	0.007	0.84	0.74 to 0.96	0.010	0.98	0.85 to 1.12	0.740
55–64	3807	0.86	0.77 to 0.96	0.007	0.90	0.80 to 1.02	0.114	0.94	0.83 to 1.08	0.417
≥65	1694	0.95	0.83 to 1.10	0.511	0.91	0.77 to 1.07	0.244	1.00	0.84 to 1.19	0.980
**Area**										
City (reference)	5585	1.00	—	—	1.00	—	—	1.00	—	—
Suburb	2676	1.00	0.89 to 1.12	0.964	1.03	0.90 to 1.16	0.680	0.87	0.76 to 1.00	0.053
Small town	2760	0.68	0.60 to 0.77	<0.001	0.78	0.68 to 0.90	<0.001	0.95	0.82 to 1.10	0.500
Rural	1783	0.75	0.65 to 0.87	<0.001	0.87	0.74 to 1.01	0.071	0.93	0.78 to 1.10	0.398
**Communication with other HCPs**										
Yes, often (reference)	8160	1.00	—	—	1.00	—	—	1.00	—	—
Sometimes or rarely	3834	1.58	1.43 to 1.74	<0.001	1.62	1.46 to 1.80	<0.001	1.95	1.74 to 2.18	<0.001
Never	125	2.26	1.53 to 3.33	<0.001	1.91	1.26 to 2.92	0.002	2.87	1.86 to 4.44	<0.001
**GP communicates using email**										
No (reference)	4660	1.00	—	—	1.00	—	—	1.00	—	—
Yes	8024	0.89	0.81 to 0.97	0.010	0.79	0.71 to 0.87	<0.001	0.87	0.76 to 0.99	0.036
**Mean consultation time**										
≤5 minutes to ≤10 minutes (reference)	2703	1.00	—	—	1.00	—	—	1.00	—	—
>10 minutes to ≤15 minutes	4241	1.73	1.50 to 2.00	<0.001	1.75	1.50 to 2.04	<0.001	1.04	0.86 to 1.27	0.662
>15 minutes to ≤20 minutes	3290	2.28	1.97 to 2.63	<0.001	2.37	2.02 to 2.78	<0.001	0.98	0.78 to 1.22	0.838
>20 minutes to ≤90 minutes	2628	2.23	1.92 to 2.59	<0.001	2.18	1.84 to 2.59	<0.001	1.05	0.83 to 1.33	0.671
**Practice type**										
Traditional (reference)	1154	1.00	—	—	1.00	—	—	1.00	—	—
Multidisciplinary	239	0.83	0.71 to 0.96	0.014	0.70	0.59 to 0.84	<0.001	0.99	0.81 to 1.21	0.930
Nurse-centred	244	0.90	0.77 to 1.05	0.179	0.82	0.69 to 0.97	0.023	0.91	0.75 to 1.10	0.320
Psychologist-centred	591	1.54	1.38 to 1.72	<0.001	1.54	1.36 to 1.75	<0.001	1.14	0.98 to 1.32	0.090
Physiotherapist-centred	182	1.72	1.44 to 2.06	<0.001	1.81	1.49 to 2.20	<0.001	1.34	1.08 to 1.67	0.009
**Country**										
Australia (reference)		1.00	—	—	—	—	—	1.00	—	—
Canada		2.10	1.51 to 2.91	<0.001	—	—	—	2.18	1.47 to 3.22	<0.001
France		11.05	7.96 to 15.35	<0.001	—	—	—	15.74	10.67 to 23.22	<0.001
Germany		0.17	0.09 to 0.32	<0.001	—	—	—	0.18	0.08 to 0.39	<0.001
The Netherlands		0.45	0.28 to 0.71	0.001	—	—	—	0.64	0.38 to 1.09	0.098
New Zealand		1.34	0.89 to 2.03	0.160	—	—	—	1.58	0.99 to 2.52	0.056
Norway		1.29	0.87 to 1.92	0.200	—	—	—	2.04	1.29 to 3.25	0.002
Sweden		3.36	2.43 to 4.65	<0.001	—	—	—	4.67	3.10 to 7.04	<0.001
Switzerland		1.46	1.02 to 2.09	0.039	—	—	—	1.49	0.96 to 2.31	0.074
UK		2.17	1.53 to 3.07	<0.001	—	—	—	2.85	1.86 to 4.37	<0.001
US		1.65	1.18 to 2.33	0.004	—	—	—	1.83	1.20 to 2.80	0.005

^a^Without adjusting for countries. ^b^Adjusting for individual countries. HCP = healthcare professional. OR = odds ratio.

## Discussion

### Summary

According to our findings, half of the practices in the 11 Western countries surveyed were of the traditional type (51.6%), characterised by the relatively small numbers of different HCPs comprising their staff. Multidisciplinary practices, characterised by significant numbers of all kinds of HCPs, represented only 11.9% of all the practices. However, those multidisciplinary practices reported better outcomes than did traditional ones regarding their coordination with SS and CPs, and their GPs’ perceived ability to manage the care of patients with chronic conditions.

### Strengths and limitations

This study had some limitations, including the low participation rate in some countries. Also, modes of participant recruitment and data collection and completion varied across countries. The self-reported nature of our data makes them subject to the possibility of self-report biases. Some definitions of HCPs may vary according to country. Also, because information about overall headcounts was unavailable, observations about practice size and its implications for the optimal distribution of the HCPs working in them were limited. Furthermore, the HCP’s location (inside or outside the practice) is not specified. What’s more, most of the results are no longer significant when adjusted to countries, reflecting the strong influence of different healthcare systems, making comparisons between the different countries in the study difficult (see Supplementary Table S2).

Finally, the data come from a study carried out in 2019, before the outbreak of COVID-19. The pandemic may have changed practice in PC in many countries, and there may have been policy changes implemented in the intervening years.

One strength of this study was the overall sample size; another was the questionnaire’s standardised methodology that enabled international comparisons. The Western countries selected were all high-income countries with similar population health outcomes that allowed comparisons.

### Comparison with existing literature

International recommendations suggest reforming PC systems so that they use the better interprofessional collaboration afforded by multidisciplinary teams to better support patients with chronic conditions.^1,40^ However, our findings revealed the great heterogeneity in the degree to which this transition in practice type has been implemented across Western countries.

Traditional, nurse-centred, and physiotherapist-centred types of PCTs were characterised by little variety in the composition of their HCPs (either MAs in traditional practices, nurses or APNs in nurse-centred practices, and physiotherapists in physiotherapist-centred practices). We hypothesised that having MAs, nurses, or APNs in a practice might restrict transitions towards multidisciplinarity (explained by the fact that MA, nurse, and APN activities can overlap with those of other HCPs) by limiting how many other HCPs were represented or ‘needed’ in these types of practices. In contrast, multidisciplinary practices, which were found predominantly in Canada and the US, comprised high percentages of different HCPs (except for MAs) and systematically included SWs. US healthcare policies could partially explain this trend as it is the only Western country that has neither mandatory health assurance nor a national healthcare system: this may have been a strong incentive for the US to set up a cost-saving healthcare system to compensate for the lack of government healthcare spending for its population. It is also interesting to note that Sweden, which reported high numbers of different HCPs in its PC practices, did not report any multidisciplinary practices (0%) because its practices never comprise SWs. In Sweden, health care and SS are separate in terms of governance and funding.^41,42^ This explains why Sweden mainly reported psychologist-centred practices (52.9%), which, like multidisciplinary practices, include relatively high proportions of different HCPs.

Finally, even though some types of practices were more or less present in the different countries, no practice type was specific to any one country or group of countries, reflecting how Western PC health systems are transitioning from traditional models towards multidisciplinarity.

Concerning associations between types of practice and their ability to manage the care of patients with chronic conditions, multidisciplinary practices reported better communication with SS and CPs than did traditional ones. Our study corroborated that communication with SS and CPs is facilitated by integrating SWs into multidisciplinary type practices. However, collaboration with SWs was unusual among traditional type practices (only 0.7% of them), despite them being the most common type of PCT. The multitasking activities of MAs or nurses, evoked above, seem unable to respond properly to patients’ social needs, and specialist SWs seem indispensable. Furthermore, only 14.7% of all the responding practices (over all the countries) worked with SWs. Strengthening and promoting practices’ collaboration with SWs seems crucial to future healthcare policies. Furthermore, communication with SS and CPs does not only depend on PCTs structuration but also depend on the structuration, organisation, and policy-related constraints of SS (for example, reimbursement mechanisms, levels of training, country or municipalities control, and human resources).

The management of patients with chronic conditions also works better through multidisciplinary practices than traditional ones. Generally speaking, practices that were ‘over-specialised’ (with a high proportion of one type of HCP, for example, as in physiotherapist-centred or psychologist-centred practices) were not as well prepared for managing patients with chronic conditions.

As with multidisciplinary practices, nurse-centred practices reported being better prepared to manage the care of patients with chronic conditions than traditional ones. The commonality between these two types of practice was the high proportion of APNs in their teams (72.7% and 89.2%, respectively) able to take on the role of case manager, which is an important role in healthcare coordination and very much needed by patients with multimorbidity.

When the types of practices characterised were adjusted by country, most of the results discussed above were no longer significant (considered an overall proxy for their national healthcare systems), a testament to how important national specificities are on these indicators. However, multidisciplinary practices did remain significantly better at communication with SS and CPs than traditional ones even after adjustment by country.

### Implications for research and practice

Despite strong arguments in favour of developing multiprofessional PCTs, most of the countries analysed have yet to adopt this type of practice as standard. However, the great variety of different practice types within countries was an indicator that healthcare systems were in transition. According to GPs, practices working with multidisciplinary teams demonstrated better overall communication (with SS, home-care services, and by email with patients) and a greater level of ability to manage the care of patients with chronic conditions. These results should encourage governments to continue efforts to transition towards multidisciplinary PC. Further research could emphasise our findings by focusing on how multidisciplinary models of care affect indicators of communication and access to care.

## Data

The data are the property of the Commonwealth Fund. We obtained them for free from the Swiss Federal Office of Public Health.
